# Advanced Oxidative Protein Products Cause Pain Hypersensitivity in Rats by Inducing Dorsal Root Ganglion Neurons Apoptosis via NADPH Oxidase 4/c-Jun N-terminal Kinase Pathways

**DOI:** 10.3389/fnmol.2017.00195

**Published:** 2017-06-19

**Authors:** Ruoting Ding, Baihui Sun, Zhongyuan Liu, Xinqiang Yao, Haiming Wang, Xing Shen, Hui Jiang, Jianting Chen

**Affiliations:** ^1^Department of Spine Surgery, Nanfang Hospital, Southern Medical UniversityGuangzhou, China; ^2^Department of Plastic and Aesthetic Surgery, Nanfang Hospital, Southern Medical UniversityGuangzhou, China

**Keywords:** advanced oxidative protein products, hypersensitivity, apoptosis, oxidative stress, dorsal root ganglion

## Abstract

Pain hypersensitivity is the most common category of chronic pain and is difficult to cure. Oxidative stress and certain cells apoptosis, such as dorsal root ganglion (DRG) neurons, play an essential role in the induction and development of pain hypersensitivity. The focus of this study is at a more specific molecular level. We investigated the role of advanced oxidative protein products (AOPPs) in inducing hypersensitivity and the cellular mechanism underlying the proapoptotic effect of AOPPs. Normal rats were injected by AOPPs-Rat serum albumin (AOPPs–RSA) to cause pain hypersensitivity. Primary cultured DRG neurons were treated with increasing concentrations of AOPPs–RSA or for increasing time durations. The MTT, flow cytometry and western blot analyses were performed in the DRG neurons. A loss of mitochondrial membrane potential (MMP) and an increase in intracellular reactive oxygen species (ROS) were observed. We found that AOPPs triggered DRG neurons apoptosis and MMP loss. After AOPPs treatment, intracellular ROS generation increased in a time- and dose-dependent manner, whereas, *N*-acetyl-L-cysteine (NAC), a specific ROS scavenger could inhibit the ROS generation. Proapoptotic proteins, such as Bax, caspase 9/caspase 3, and PARP-1 were activated, whereas anti-apoptotic Bcl-2 protein was down-regulated. AOPPs also increased Nox4 and JNK expression. Taken together, these findings suggest that AOPPs cause pain hypersensitivity in rats, and extracellular AOPPs accumulation triggered Nox4-dependent ROS production, which activated JNK, and induced DRG neurons apoptosis by activating caspase 3 and PARP-1.

## Introduction

Pain hypersensitivity, a widespread and highly debilitating condition with which clinical treatment remains challenging, is the most common category of chronic pain. It may be triggered or initiated by a primary lesion or dysfunction of the somatosensory nervous system (Staaf et al., 2009). Because of its elusive cellular and physiopathologic mechanisms, hypersensitivity is difficult to cure (Chen et al., 2015). A lot of evidence indicates that oxidative stress and certain cells apoptosis, such as dorsal root ganglion (DRG) neurons, have a role in the induction and development of hypersensitivity (Jiang et al., 2008; Huang et al., 2014).

Oxidative stress occurs when the antioxidant capacity is decreased and excessive amount of reactive oxygen species (ROS) is formed. Relevant studies reported that nicotinamide adenine dinucleotide phosphate (NADPH) oxidase (Nox) pathway and mitochondrial respiratory cycle are two main sources of cellular ROS (Ozdemir et al., 2015). In particularly, Nox4 has been studied to play a significance role in the ROS-dependent activation in DRG neurons (Im et al., 2012). Additional evidence suggests that the DRG neurons are greatly sensitized to ROS damage due to the blood–nerve and perineurial barriers are low at this site (Kahya et al., 2016). Meanwhile, mitochondria are vulnerable to ROS attack. ROS cause oxidative damage to mitochondrial membrane integrity and DNA, leading to loss of mitochondrial membrane potential (MMP) and activation of DNA repair proteins. ROS also mediate the mitogen-activated protein kinase (MAPK) pathway and induce cell apoptosis. Mitochondrial damage and MAPK pathway both can trigger cell apoptosis (Ott et al., 2007).

Reactive oxygen species have been suggested as the main cause of neurons apoptosis and several antioxidants and radical scavengers can alleviate cell death *in vitro* and *in vivo* (Naziroglu, 2012; Balaban et al., 2016). Apoptosis is a process of programmed cell death. Physiological cell death that removes unwanted cells plays an important role in the development, tissue homeostasis and defense against viral infection and mutation. Disordered apoptosis is implicated in a variety of pathologies, including hypersensitivity (Hu et al., 2015). In recent studies, apoptotic neuronal subpopulations undergoing apoptosis show an increased Bax and Caspase 3 gene expression. Caspases are a family of intracellular cysteine proteases and are famous for their roles in management of apoptosis and neurodegeneration (Unsain et al., 2013; Berta et al., 2014).

Recent studies have proposed that oxidative stress can cause pain hypersensitivity (Kallenborn-Gerhardt et al., 2013). Laboratory researches have detected the accumulation of many oxidative products in patients with hypersensitivity. Also, many studies have demonstrated the existence of advanced oxidative protein products (AOPPs) (Moon et al., 2015; Griggs et al., 2016). AOPPs are dityrosine-containing and cross-linking protein products formed primarily as a consequence of oxidative stress. They participate, by inducing cell apoptosis, in the pathogenesis of many diseases. Furthermore, the roles of AOPPs in activating NADPH oxidase have been well documented (Wei et al., 2009). However, the fact of if and how AOPPs induce DRG neurons apoptosis has not been investigated yet.

Therefore, this study was conducted to examine whether AOPPs could cause hypersensitivity via inducing DRG neurons apoptosis *in vivo* and *in vitro*. Herein, we tested the DRG neurons apoptosis in AOPPs-induced pain hypersensitivity rats. Also, this study determined the effects of AOPPs on primary cultured DRG neurons apoptosis and investigated the molecular mechanism underlying the proapoptotic effect of AOPPs.

## Materials and Methods

### Animals

Twenty-five Male Sprague-Dawley rats (initial age 8 weeks, initial weight 200–250 g, Animal Experiment Center, Southern Medical University, Guangzhou, China) were used in this study. Rats were housed 5 per cage under a standard 12 h/12 h light/dark cycle with food and water available ad libitum. Before any experimental procedures, rats were acclimated for 7 days. All experiments followed the recommendations of the International Association of Studies on Pain, and were approved by the Laboratory Animal Care and Use Committee of Nanfang hospital, Southern Medical University (NFYY-2014-86).

### Animal Experimental Design

The rats were randomly divided into five groups (five rats per group): AOPPs-Rat serum albumin (AOPPs–RSA) group, PBS group, Rat serum albumin (RSA) group, apocynin group, and AOPPs–RSA+apocynin group. In our study, we injected AOPPs–RSA into healthy rats, according to plasma AOPPs concentration in the CFA model and our previous study (Ding et al., 2016). AOPPs–RSA (50 mg/kg), RSA (50 mg/kg), and PBS (pH 7.4) were intravenously injected each day of the study, Antioxidant apocynin (50 mg/kg, Sigma–Aldrich Corp, MO, United States) was dosed by intragastric administration each day of the study duration (Ding et al., 2016). The basic threshold of each rat was tested 3 days before drugs were injected. After treatment, rats were tested on Days 0, 3, 6, 9, ....., 54, and 57. The time interval was 2 days. DRG tissues were obtained after deeply anesthetized with pentobarbital sodium (150 mg/kg) on Day 57.

### Preparation of AOPPs–RSA and Determination

As previously described, AOPPs-Rat Serum Albumin (RSA) was prepared *in vitro* by incubating RSA (Sigma–Aldrich Corp, MO, United States) with hypochlorous acid (HOCl) as described in a previous study (Ding et al., 2016). The samples were dialyzed for 24 h against PBS at 4°C to remove the free HOCl. Control incubation was performed in native RSA dissolved in PBS alone. All the preparations were passed through a Detoxi-Gel column (Thermo Fisher Scientific, MA, United States) to remove any contaminated endotoxin. To quantify AOPPs concentration, SpectraMax M5 Multifunctional Microplate Reader (Molecular Devices, CA, United States) was used to measure the absorbance at 340 nm immediately.

### Mechanical Hypersensitivity Assays

This study was performed at the same time of day and by an experimenter unaware of the treatment applied. Each rat was placed in a clear plastic chamber on an elevated wire grid and acclimated for at least 15 min to the testing environment prior to the experiment. Withdrawal responses to mechanical stimuli were determined using an Electronic von Frey Anesthesiometer system (IITC Life Science, CA, United States). The electronic von Frey polypropylene tip was applied perpendicularly to the mid-plantar surface of the selected hind paw and the intensity of the stimulus was automatically recorded when the paw was flexed reflexively followed by a clear flinch response after paw withdrawal. All rats were tested 5 times, with an inter-test period of 15 min (Shibasaki et al., 2010).

### Immunofluorescence Staining

Immunofluorescence staining of DRG slides was performed as previously described (Morell et al., 2014). Briefly, rats were anesthetized with isoflurane and pentobarbital sodium (150 mg/kg) at Day 57. The lumber DRGs (L4–6) were removed and postfixed in 4% paraformaldehyde overnight at 4°C and then were transferred into 30% sucrose (in PBS) at 4°C for at least 24 h. Samples were cryostat sectioned at a thickness of 14–16 μm and stored at –80 °C until use. Sections were permeabilized, blocked in 5% bovine serum albumin in PBS for 1 h at room temperature and then incubated overnight at 4°C with the following antibodies: rabbit anti-Nox4 (1:500) and chicken anti-beta III Tubulin antibody (1:1000) from Abcam (MA, United States). After rinsing in PBS, slides were incubated with secondary antibodies conjugated with FITC (1:500) or Cy3 (1:200) for 2 h at room temperature. Then, reacted slides were mounted and cover slipped. Images were captured with an Olympus FluoView FV10i self-contained confocal laser scanning microscope system (Olympus America Inc., PA, United States).

### TUNEL Analysis

To determine whether cell apoptosis occurs in the primary afferent neurons during AOPPs-induced pain hypersensitivity rats, we used terminal deoxynucleotidyl transferase-mediated dUTP nick end-labeling (TUNEL) assay to evaluate programmed cell death in the dorsal root ganglia (location of the cell bodies of the primary afferent neurons) in each group of rats. Rats were deeply anaesthetized with sodium pentobarbital (150 mg/kg) and the lumber DRGs (L4–6) were removed and postfixed in 4 % paraformaldehyde overnight at 4°C. Cryosections were evaluated for TUNEL staining using the *In situ* Cell Death Detection Kit, Fluorescein (Roche) according to the manufacturer’s instructions (Ben-Zvi et al., 2008).

### Primary Sensory Neurons Culture

Dorsal root ganglion from thoracic and lumbar spinal cord of rats were minced in cold PBS and incubated for 60–90 min at 37°C in DMEM (4.5 g/L glucose, Gibco) containing (in mg/mL): 0.5 trypsin, 1 collagenase type IA, and 0.1 DNase type IV. Fetal bovine serum was added to neutralize trypsin. Neurons were pelleted, suspended in DMEM containing 10% fetal bovine serum, 100 U/mL penicillin, 0.1 mg/mL streptomycin, 2 mM glutamine. Cells were plated on polylysine-coated wells. Cytosine arabinoside (1 μM) was added to eliminate mitotic cells, which including Schwann cells and fibroblasts. Cultures were maintained at 37°C in a water-saturated atmosphere with 5% CO_2_ for 3 d prior to start of the experiment (Amadesi et al., 2004).

### Assessment of Apoptosis by Fluorescence-Activated Cell Sorting

Dorsal root ganglion neurons were cultured at 75% confluence and treated with AOPPs–RSA at increasing concentrations for 24 h or 100 μg/mL AOPPs–RSA for increasing time durations. After treatment, the cells were resuspended in 500 μL of binding buffer containing 5 μL Annexin V–fluorescein isothiocyanate (FITC) and 3 μL of propidium iodide (PI) according to the manufacturer’s instructions (BD Biosciences, CA, United States), and incubated for 10 min in the dark. Quantification of Annexin V–FITC and PI staining was performed by FACSCanto II (BD Biosciences) using channels FL-1 (Annexin V–FITC) and FL-3 (PI).

### Small Interfering RNA (siRNA) Transfection

To introduce siRNA into rat DRG neurons, the cells were plated on 6-well plates at 90% confluence before transfection. Individual siRNAs (at 5 nM), lipofectamine 3000 and Opti-MEM were mixed and incubated at room temperature for 20 min. siRNA– lipofectamine 3000 complexes were added to cells for 24 h and the medium was replaced by fresh serum DMEM medium after transfection. Nox4 siRNA (190243) were purchased from Thermo Fisher Scientific (MA, United States). Experiments were performed 72 h after transfection. Knockdown of Nox4 was assessed by Western blot analysis which detected the protein level of Nox4 (Liu et al., 2016).

### Determination of ROS Generation

The level of intracellular ROS was assessed by fluorescence microplate reader and confocal laser scanning microscope system (Olympus America Inc., PA, United States) with the probe 2′,7′-dichlorofluorescein diacetate (DCFH-DA), which oxidizes to fluorescent dichlorofluorescein (DCF) in the presence of ROS, as described previously (Ding et al., 2016). Briefly, DRG neurons were suspended in DMEM at a given concentration of 10^8^/L. Each sample was incubated in 10 μM DCFH-DA (Sigma–Aldrich Corp, MO, United States) for 30 min in darkness. The excitation and emission wavelengths were 488 and 525 nm, respectively. The resulting data were normalized using the control values.

### Assessment of the Change in MMP

The change in MMP caused by AOPPs was assayed using a membrane-permeant JC-1 dye (5,50,6,60-tetrachloro-1,10,3,30-tetraethylbenzimidazolylcarbocyanine iodide; Keygen Biotech, Nanjing, China). The JC-1 dye accumulates in mitochondria in a potential-dependent manner. Consequently, mitochondrial depolarization is indicated by a decrease in the red/green fluorescence intensity ratio. The potential-sensitive color shift is due to concentration-dependent formation of red fluorescent J-aggregates (Sun et al., 2016). After 24 h of AOPPs–RSA treatment, 5 μg/mL of JC-1 dye was added to the cells with incubation at 37°C for 20 min, and the cells were washed with incubation buffer. The alteration in DRG neurons was observed using the Olympus FluoView FV10i self-contained confocal laser scanning microscope system.

### Western Blot Analysis

Samples were homogenized in ice-cold RIPA buffer with 1 mM PMSF, protease, and phosphatase inhibitors (Sigma–Aldrich Corp., MO, United States) and cleared by centrifugation (12 000 rpm, 4°C, 10 min). Equivalent amounts of extracted proteins (50 μg) were separated by 10 or 12% SDS–polyacrylamide gel electrophoresis, then electro-blotted onto polyvinylidene difluoride membranes (Millipore, MA, United States). The membranes were blocked using 5% blocking buffer for 1 h at room temperature. Blots were incubated overnight at 4°C with the following primary antibodies: rabbit anti-Nox4 (1:1000) from Abcam (MA, United States), rabbit anti-(*p*-)MAPK family (1:1000), rabbit anti-Bax (1:1000), rabbit anti-Bcl2 (1:1000), rabbit anti-Cytochrome c (1:1000), mouse anti-caspase 9 (1:1000), rabbit anti-cleaved-caspase 9 (1:1000), rabbit anti-caspase 3 (1:1000), rabbit anti-cleaved-caspase 3 (1:1000), rabbit anti-PARP (1:1000), and rabbit anti-cleaved-PARP (1:1000) from Cell Signaling Technology (CST, MA, United States). The membrane was washed 3 times with TBST for 10 min and followed by incubation with appropriate secondary antibody for 2 h, and then washed again three times with TBST. Relative levels of immunoreactivity were quantified using the Kodak *In vivo* Imaging System (Kodak, NY, United States). Rabbit anti-β-actin (1:2000, Abcam) was used as an internal control for the concentration of proteins loaded.

### Statistical Analysis

Analyses were performed with SPSS 20.0 (IBM, NY, United States) and GraphPad Prism 5 (GraphPad Software, CA, United States) software. All experiments were repeated at least three times. Continuous variables were expressed as mean ± standard deviation (SD). Comparisons were analyzed with student *t*-test (2 groups only) or ANOVA followed by the Bonferroni *post hoc* tests analysis. A value of *P* < 0.05 was considered statistically significant.

## Results

### AOPPs Caused Mechanical Hypersensitivity through Activating Nox4 and Inducing DRG Neurons Apoptosis *In Vivo*

**FIGURE 1:**
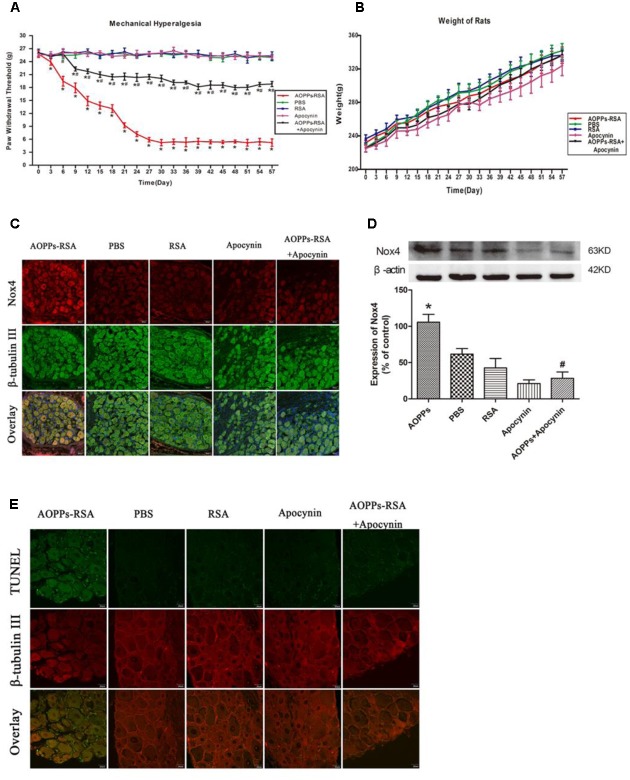
Advanced oxidative protein products (AOPPs) caused mechanical hypersensitivity through activating Nox4 and inducing dorsal root ganglion (DRG) neurons apoptosis *in vivo*. **(A)** Paw mechanical threshold was tested by the electronic von Frey system after different treatments. **(B)** The weight of each rat was measured every 3 days. **(C)** Immunofluorescence staining of DRGs in different groups, Nox4 was stained green, while β-tubulin III was red, scale bar, 40 μm. **(D)** Expression levels of Nox4 in DRGs tissues (L4–L6) were detected by Western blotting. **(E)** TUNEL assay in DRG neurons, scale bar, 20 μm. Data represent mean ± SEM of at least three independent experiments. *n* = 5 rats per group. ^∗^*P* < 0.05 versus PBS group. ^#^*P* < 0.05 versus AOPPs–RSA group.

To explore whether AOPPs could induce mechanical hypersensitivity in rats, the electronic von Frey was used to measure the mechanical threshold of rats. As shown in **Figure 1A**, after giving different treatments, the paw mechanical threshold of each rat was tested every 3 days. After intravenous injection of AOPPs–RSA, the mechanical threshold significantly declined from Day 3 (23.91 ± 0.51 g, *P* < 0.05) to Day 30 (5.22 ± 0.33 g, *P* < 0.05) and remained a low level until Day 57 (5.22 ± 0.13 g, *P* < 0.05). In addition, we found that intragastric administration of apocynin could notably improve mechanical hypersensitivity induced by AOPPs. The weight of rats in each group was recorded in **Figure 1B**, and there was no significant difference between each group (the range was from 225.00 ± 4.21 g to 341.80 ± 8.46 g, *P* > 0.05).

Immunoreactivity of anti-Nox4 was observed in sections of rat DRG (L4-L6) neurons that were co-stained with the neuronal marker, β-tubulin III. **Figure 1C** revealed that Nox4 was markedly increased in AOPPs–RSA group compared with that in PBS group, and apocynin partly inhibited AOPPs-induced Nox4 expression. To confirm our results, we used western blot assays to study the expression level of Nox4 in DRG tissues (L4-L6). **Figure 1D** indicated that the expression of Nox4 increased ∼2-fold in AOPPs–RSA group compared with PBS group (*P* < 0.05), and apocynin could reduce the expression level by almost 75% (*P* < 0.05).

We performed TUNEL assay in order to evaluate the occurrence of apoptosis in DRG neurons of AOPPs-induced rats. The results from this study showed the evidence of programmed cell death or of necrotic cell death in the DRG of AOPPs-induced rats (**Figure 1E**). These studies indicate that cell apoptosis was occurred in the DRG neurons. Taken collectively, our data indicate that AOPPs could activate Nox4 expression, induce apoptosis of DRG neurons, finally lead to causing pain hypersensitivity in rats.

### AOPPs-Induced Primary DRG Neurons Apoptosis

**FIGURE 2:**
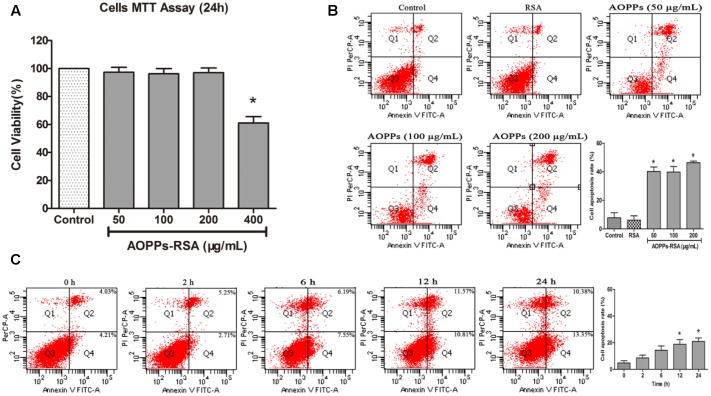
Advanced oxidative protein products -induced DRG neurons apoptosis. **(A)** Viability of DRG neurons treated with AOPPs at different concentrations. **(B)** Representative dot blots of Annexin V–FITC versus PI. Primary DRG neurons were incubated with AOPPs–RSA for the indicated concentrations or unmodified RSA for 24 h. **(C)** DRG neurons were incubated with 100 μg/mL AOPPs–RSA for the indicated time durations. Apoptosis was quantified by measuring the number of combined early and late apoptotic cells using flow cytometry and was found to increase in a concentration and time-dependent manner. Data represent mean ± SEM of at least three independent experiments. ^∗^*P* < 0.05 versus control group (or time = 0).

The MTT assay (**Figure 2A**) showed that incubation in 0–200 μg/mL AOPPs–RSA had minimal effects on cell fatality (cell viability was above 90%, *P* > 0.05). To eliminate the fatal effects of AOPPs, DRG neurons were subjected to increasing concentrations of AOPPs–RSA (50, 100, and 200 μg/mL) for 24 h. Quantitative fluorescence-activated cell sorting analysis of FITC–annexin V/PI staining indicated that AOPPs–RSA induced DRG neurons apoptosis (**Figure 2B**, *P* < 0.05). **Figure 2C** showed that 100 μg/mL AOPPs–RSA for 12 and 24 h could significantly induce apoptosis of DRG neurons (*P* < 0.05).

### Caspase Cascade and PARP-1 were Activated in AOPPs-Treated Primary DRG Neurons

**FIGURE 3:**
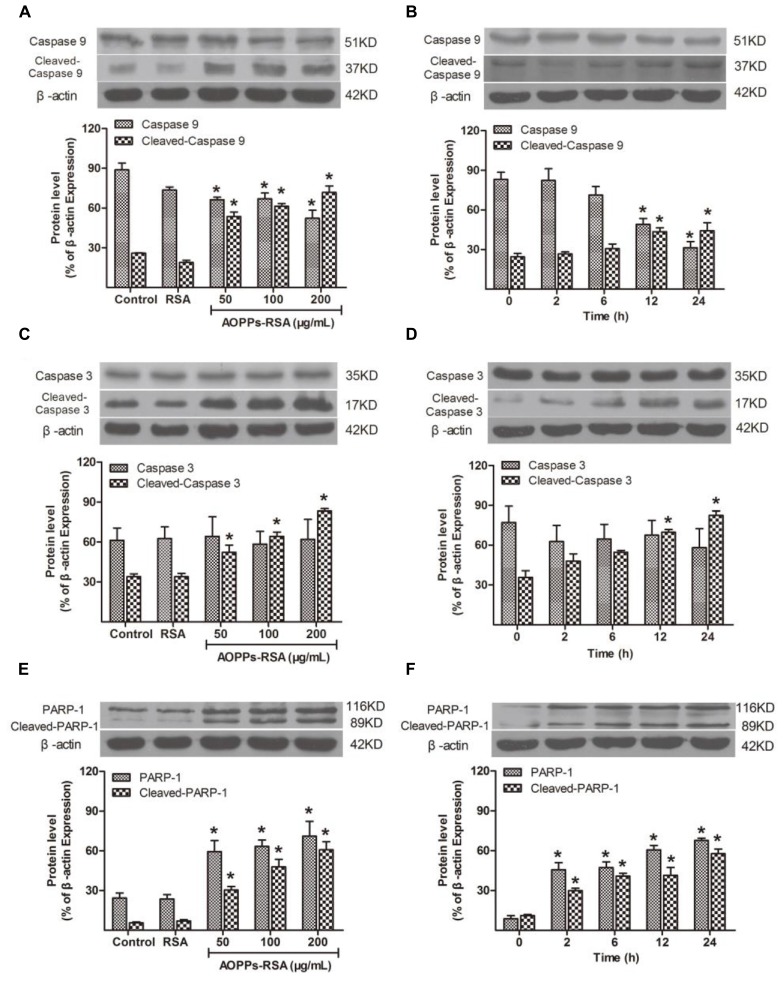
Apoptosis-related cellular events after AOPPs–RSA treatment. **(A,B)** Pro-caspase 9 significantly decreased from 12 h, accompanied by an overexpression of cleaved-caspase 9 in a dose-dependent manner. **(C,D)** Cleaved-caspase 3 increased from 12 h, however, pro-caspase 3 had no significant changes. **(E)** PARP-1 and cleaved-PARP-1 activation were enhanced by AOPPs–RSA at increasing concentrations. **(F)** PARP-1 and cleaved-PARP-1 increased from 2 h and reached the highest level at 24 h 05). Data represent mean ± SEM of at least three independent experiments. ^∗^*P*<0.05 versus control group (or time = 0).

The caspase family is a key section in cell apoptosis, and activation of caspase cascade and PARP-1 results in substantial damage in molecule level. To demonstrate whether caspase cascade and PARP-1 participate in AOPPs-induced DRG neurons apoptosis, their expression was detected by western blot analysis. The expression of pro-caspase9 decreased in DRG neurons under the condition of 50, 100, or 200 μg/mL AOPPs–RSA in 24 h, while, cleaved-caspase9 increased significantly (**Figure 3A**, *P* < 0.05). Next, we used 100 μg/mL AOPPs–RSA to treat cells in indicated periods, the results indicated that the levels of pro-caspase9 was significantly down-regulated at 12 and 24 h, whereas, cleaved-caspase9 was up-regulated (**Figure 3B**, *P* < 0.05). As for cleaved-caspase3, the expression level was similar to cleaved-caspase9. However, the level of pro-caspase3 had no significant changes in indicated concentrations or periods (**Figures 3C,D**, *P* > 0.05). PARP-1 and cleaved-PARP-1 increased in AOPPs-treated cells (**Figure 3E**, *P* < 0.05), and the level of protein increased from 2 h after AOPPs–RSA treatment and reached the highest level at 24 h (6.57-fold and 5.76-fold compared with *t* = 0, *P* < 0.05, **Figure 3F**).

### AOPPs–RSA Induced Mitochondrial Dysfunction in Primary DRG Neurons

**FIGURE 4:**
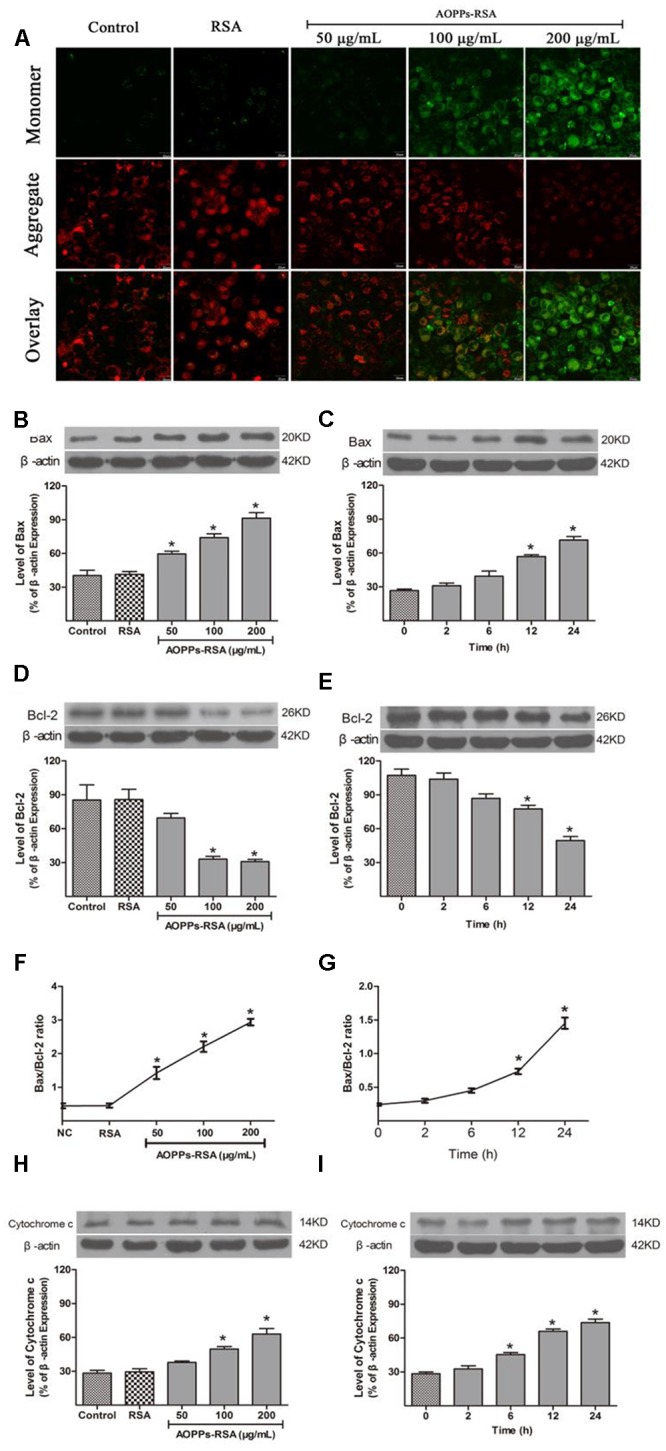
Advanced oxidative protein products–RSA induced mitochondrial dysfunction in primary DRG neurons. **(A)** The mitochondrial membrane potential (MMP) was observed in AOPPs-treated DRG neurons. The cells were incubated with 100 μg/mL AOPPs–RSA for 24 h and then JC-1 dye for 20 min. The cells were observed using a confocal laser scanning microscope system and demonstrated an AOPPs-dependent MMP loss in a dose-dependent manner, scale bar, 20 μm. **(B–G)** Proapoptotic Bax increased and antiapoptotic Bcl-2 decreased from 12 h; prolonged incubation and a higher concentration of AOPPs–RSA increased the Bax/Bcl-2 ratio. **(H,I)** Cytochrome c increased in AOPPs-treated DRg neurons. Data represent mean ± SEM of at least 3 independent experiments. ^∗^*P* < 0.05 versus control group. ^#^*P* < 0.05 versus AOPPs–RSA group.

Loss of mitochondrial membrane potential (MMP) was considered as a form of mitochondrial dysfunction. MMP loss was assessed through JC-1 staining. In healthy cells with a high MMP, JC-1 accumulates in the mitochondria as J-aggregates with red fluorescence. As the MMP declines in apoptotic cells, JC-1 stays in the cytosol as monomers with green fluorescence. The results suggested a dose-dependent reduction of red fluorescence and concomitantly enhanced green fluorescence, which indicated the existence of MMP loss in AOPPs-treated DRG neurons (**Figure 4A**).

The intrinsic apoptosis pathway is regulated primarily by Bcl-2 family proteins, notably the proapoptotic Bax and antiapoptotic Bcl-2 proteins. **Figure 4B** showed that the expression of Bax was activated in a concentration-dependent manner, while Bcl-2 was down-regulated (**Figure 4D**), and the highest Bax/Bcl-2 ratio was 2.93 ± 0.15 at the concentration of 200 μg/mL AOPPs–RSA (**Figure 4F**, *P* < 0.05). As shown in **Figures 4C,E**, the expression of proapoptotic Bax significantly increased from 12 h, with a significantly decrease in antiapoptotic Bcl-2 at the same time, and the highest Bax/Bcl-2 ratio was 1.45 ± 0.12 at 24 h (**Figure 4G**, *P* < 0.05).

Cytochrome c increased in cytoplasm when mitochondrial membrane potential decreased, and it can induce apoptosis of cells. As shown in **Figures 4H,I**, cytochrome c increased significantly after AOPPs–RSA treated DRG neurons in indicated concentrations or periods.

### AOPPs Activated JNK via Nox4–ROS Pathway

**FIGURE 5:**
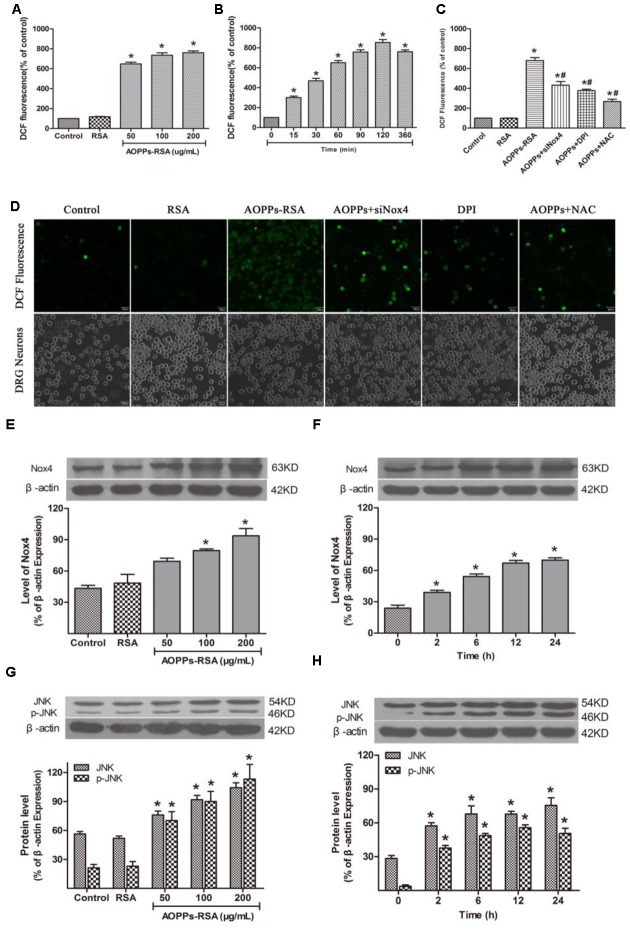
Advanced oxidative protein products activated JNK via Nox4–ROS pathway. **(A)** DRG neurons were incubated in AOPPs–RSA, unmodified RSA, or control medium for 6 h prior to DCFH-DA (10 μM) treatment. **(B)** DRG neurons were incubated in 200 μg/mL AOPPs–RSA for indicated time durations followed by DCFH-DA treatment. **(C)** DRG neurons were treated with AOPPs–RSA (200 μg/mL) with or without Nox4 siRNA, DPI (10 μM, 2 h) or NAC (2 mM, 2 h). **(D)** DRG neurons were cultured with control medium, RSA, AOPPs–RSA (200 μg/mL), AOPPs+siNox4, AOPPs+DPI, or AOPPs+NAC for 360 min. Confocal laser scanning microscope system was used to visualize ROS generation in DRG neurons with the use of DCFH-DA, scale bar, 30 μm. **(E,F)** The level of Nox4 was up-regulated significantly in DRG neurons incubated with AOPPs–RSA in indicated concentrations or periods. **(G,H)** The expression level of JNK/p-JNK. Data represent mean ± SEM of at least three independent experiments. ^∗^*P* < 0.05 versus control (0) group. ^#^*P* < 0.05 versus AOPPs–RSA group.

To determine whether AOPPs, as oxidative stress products, can cause abundant ROS accumulation inside DRG neurons, we examined intracellular ROS levels in AOPPs-treated DRG neurons. We subjected DRG neurons cultures to increasing concentrations of AOPPs–RSA or for different time durations. ROS production was increased in DRG neurons cultured with AOPPs–RSA in a dose- and time-dependent manner (**Figures 5A,B**). To elucidate the function of Nox4 in ROS generation, DRG neurons were, respectively, pre-treated with Nox4 siRNA (Supplementary Figure S1), Nox inhibitor DPI (10 μM, 2 h) and ROS scavenger NAC (2 mM, 2 h). After these treatments, DRG neurons were incubated with 200 μg/mL AOPPs–RSA for 360 min. The level of ROS generation was significantly decreased in DRG neurons that were pretreated with Nox4 siRNA, DPI or NAC separately (**Figure 5C**). For further confirmation, ROS generation in DRG neurons cultured with control medium, RSA, AOPPs–RSA, AOPPs+siNox4, AOPPs+DPI, AOPPs+NAC was observed under a confocal laser scanning microscope system using DCFH-DA (**Figure 5D**).

To investigate the effect of AOPPs–RSA on Nox4 expression in rat DRG neurons, the cells were stimulated with different concentrations of AOPPs–RSA (0, 50, 100, and 200 μg/mL) or for different time durations (0, 2, 6, 12, and 24 h). The protein level of Nox4 was analyzed by western blot from AOPPs-stimulated rat DRG neurons. The Nox4 protein level gradually increased after AOPPs stimulation for 100–200 μg/mL (**Figure 5E**). AOPPs–RSA stimulation also time-dependently induced Nox4 protein expression (**Figure 5F**).

Previous studies had demonstrated that ROS could activate MAPK family, thereby, lead to cell apoptosis. In this study, c-Jun N-terminal kinase (JNK), and p-JNK consecutively increased from 2 to 24 h (**Figure 5G**). Their activation was also mediated by AOPPs in a dose-dependent manner (**Figure 5H**). However, p38/p-p38 and p44/p42 had no significant changes in the process of AOPPs-induced DRG neurons apoptosis (Supplementary Figure S2).

### AOPPs–RSA Induced DRG Neurons Apoptosis via Nox4-JNK–Caspase Cascade–PARP Pathway

**FIGURE 6:**
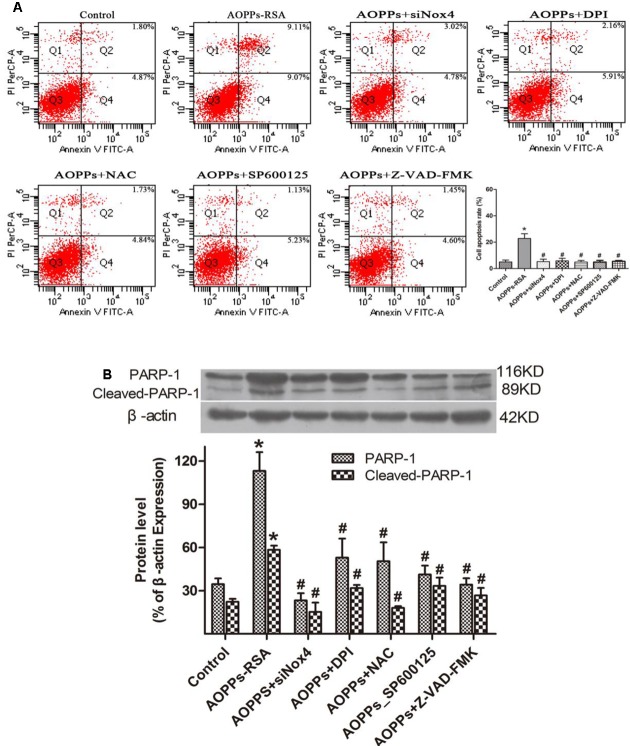
Advanced oxidative protein products-induced DRG neurons apoptosis was mediated through the Nox4–JNK–caspase cascade–PARP pathway. **(A)** DRG neurons were pre-incubated with siRNA Nox4, DPI (10 μM), NAC (2 mM), an JNK inhibitor (SP600125, 10 μM) or the broad-spectrum caspase inhibitor Z-VAD-fmk (20 μM) before AOPPs–RSA treatment. They all inhibited AOPPs-induced cell apoptosis. **(B)** The expression of PARP-1/ cleaved-PARP-1 were tested by western blots. Data represent mean ± SEM of at least three independent experiments. ^∗^*P* < 0.05 versus control group. ^#^*P* < 0.05 versus AOPPs–RSA group.

To further elucidate the roles of Nox4, ROS, JNK, caspase cascade, and PARP in AOPPs-induced apoptosis, DRG neurons were incubated with siRNA Nox4, DPI, NAC, a JNK special inhibitor (SP600125), and the broad-spectrum caspase inhibitor Z-VAD-FMK before AOPPs–RSA treatment. DRG neurons apoptosis was found to be significantly abolished under the protection of these inhibitors (**Figure 6A**) and PARP-1 activation was suppressed by these inhibitors (**Figure 6B**).

## Discussion

The present study identified that AOPPs could induce mechanical hypersensitivity *in vivo*, and AOPPs triggered DRG neurons apoptosis through a loss of MMP, enhanced expression of Bax, and reduced expression of Bcl-2. This indicated that an activation of the intrinsic mitochondrial pathway was involved in AOPPs-induced DRG neurons apoptosis. After AOPPs treatment, caspase cascade and PARP-1 were activated in DRG neurons in a time- and dose-dependent manner.

Pain hypersensitivity is a multifactorial situation caused by injury or dysfunction of the nervous system, this situation results in the increasing response to noxious stimuli (Wu et al., 2013). Our previous study indicated that AOPPs increased in complete Freund’s adjuvant (CFA)-induced hypersensitivity which is a classical model (Ding et al., 2016). In this study, we wondered whether AOPPs could induce mechanical hypersensitivity via DRG neurons apoptosis. Indeed, the paw mechanical threshold showed a tremendous decline from Day 3 to Day 30 and remained at low levels until Day 57 in AOPPs-induced rats. These data further reinforced the hypothesis of a potential role for AOPPs in the hypersensitivity processes. Interestingly, many patients suffer from hypersensitivity almost accompany with oxidative stress when excessive amount of ROS is formed or when the antioxidant capacity is decreased, such imbalance may be a key factor to pain hypersensitivity (Ibi et al., 2008; Linley et al., 2012).

The first report of AOPPs was made after the discovery of AOPPs in the plasma of uremic patients receiving maintenance dialysis (Witko-Sarsat et al., 1996). Increasing evidences suggested that AOPPs can induce the apoptosis of monocytes (Witko-Sarsat et al., 2003), intestine epithelial cells (Xie et al., 2014), chondrocytes (Wu et al., 2016) and HaCaT cells (Sun et al., 2016) leading to chronic renal failure, inflammatory bowel disease, osteoarthritis and impaired wound healing, respectively. A growing body of evidence indicates that apoptosis of DRG neurons play an important role in the development of pain hypersensitivity (Garraway et al., 2014; Mincheva-Tasheva et al., 2014; Kahya et al., 2016). In this study, we used TUNEL assay to test the apoptosis of DRG neurons in AOPPs-treated rats with hypersensitivity, and the result confirmed DRG neurons apoptosis after AOPPs-treatment. Besides, the primary DRG neurons apoptosis which were induced by AOPPs were tested by fluorescence-activated cell sorting *in vitro*. Above mentioned results revealed that apoptosis of DRG neurons, especially those diameter less than 30 μm, had a close relationship with AOPPs-induced pain hypersensitivity.

Additionally, the intrinsic apoptosis pathway was activated by AOPPs in DRG neurons. Bcl-2 family proteins, markedly the proapoptotic Bax and antiapoptotic Bcl-2 proteins primarily regulated the pathway. Overwhelming ROS generation caused Bax activation, which led to mitochondrial damage and Bcl-2 consumption (Merry et al., 1994), eventually triggering the activation of initiator (caspase 9) (Fujita et al., 2001) and effector (caspase 3) (Ferrier et al., 2015)caspases. The activated caspase 3 then induced PARP activation leading to apoptotic responses at the DNA level (Ilnytska et al., 2006). These results showed that AOPPs could activate the typical apoptosis pathway by producing a large number of ROS, cause DRG neurons apoptosis. Next, we investigate the source of ROS in the AOPPs-induced DRG neurons.

As for the source of ROS, we tested mitochondrial function and Nox expression. Mitochondrial dysfunction causes ROS overproduction. Meanwhile, mitochondria are more vulnerable to ROS attack and associated oxidative damage (Hseu et al., 2015). Loss of MMP is an early marker of mitochondrial dysfunction and also considered as a notable indicator of cell death program (Chipuk et al., 2006). Notably, Nox enzymes are distinct from most other ROS sources in that ROS generation is their major function and not a byproduct of other biological reactions. In general, 4 rodent genes of the catalytic subunit Nox (Nox1–Nox4) have been identified, which are expressed in a tissue-specific manner (Suzuki et al., 2014). For example, Nox1 is a source of ROS implicated in the development of thermal hypersensitivity (Ibi et al., 2008). Nox2 is induced in spinal cord microglia cells after peripheral nerve injury, which contributes to neuropathic pain hypersensitivity (Choi et al., 2013). Nox3 is a special one which exists in the inner ear of mice and plays a crucial role in balance and gravity (Sumimoto et al., 2005). And Nox4 contributes to pain signaling after peripheral nerve injury. Nox4 mRNA has been detected in dorsal root ganglia, and Nox4-derived ROS production in the injured peripheral nerve stump essentially contributes to the initiation or maintenance of pain hypersensitivity (Im et al., 2012). In the present study, after AOPPs–RSA treatment, Nox4 significantly increased at 2 h. Nox4 outstandingly increased after incubation with 100 μg/mL AOPPs–RSA. For further affirmed, AOPPs-induced DRG neurons apoptosis and ROS production were both inhibited by siRNA Nox4 transfection and Nox inhibitor (DPI), which demonstrated that AOPPs-induced cell apoptosis was regulated by Nox4-dependent ROS production.

Mitogen-activated protein kinase are important components involved in regulating cell apoptosis elicited by an increase in intracellular ROS (Pearson et al., 2001). In the present study, JNK and p-JNK were found to continually increase with prolonged AOPPs stimulation, they were activated most significantly under the treatment of 50 μg/mL AOPPs–RSA. By using JNK inhibitor (SP600125), DRG neurons were protected from AOPPs stimulation. However, ERK 1/2 and p-38 MAPK activation were not detected in AOPPs-treated DRG neurons. This indicated that JNK transduced a proapoptotic signal in AOPPs-treated DRG neurons and p38 MAPK and ERK1/2 might not take part in the cellular events triggered by AOPPs. Thus, MAPK family may play different roles in apoptosis in different cell types and experimental models.

**FIGURE 7:**
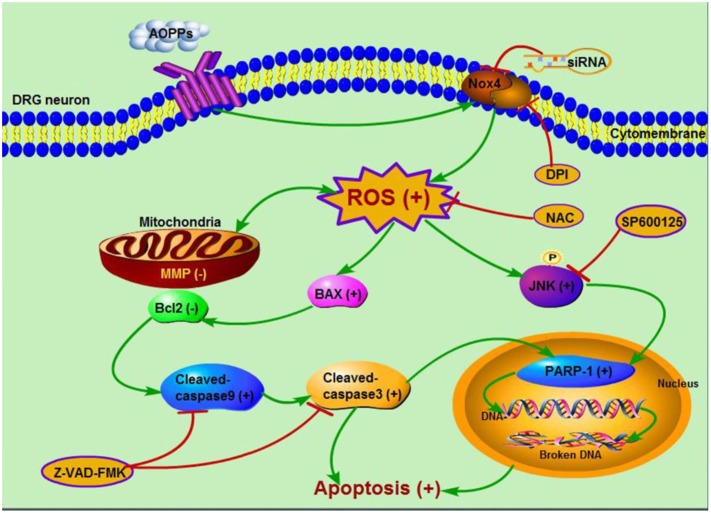
Schematic representation of AOPPs-induced DRG neurons apoptosis.

In summary, our study provided for the first time that AOPPs induced sustained mechanical hypersensitivity via DRG neurons apoptosis. Accumulation of AOPPs triggered Nox4-dependent ROS production, which activated JNK and induced DRG neurons apoptosis through caspase 9-caspase 3 and PARP-1 activation (**Figure 7**). These results indicated that AOPPs as an indicator of oxidative stress and a source of ROS played an important role in pain hypersensitivity rats, this might provide us a new way to understand the mechanism of pain hypersensitivity and give some suggestions on the therapy.

## Ethics Statement

This study was carried out in accordance with the recommendations of the International Association of Studies on Pain. The protocol was approved by the Laboratory Animal Care and Use Committee of Nanfang hospital, Southern Medical University (NFYY-2014-86).

## Author Contributions

RD and BS contributed equally to this work, they all conducted the cell research. ZL conducted animal study. XY conducted behavioral research. HW conducted TUNEL analysis. XS conducted immunofluorescence staining. HJ and JC was Corresponding author and provided guidance.

## Conflict of Interest Statement

The authors declare that the research was conducted in the absence of any commercial or financial relationships that could be construed as a potential conflict of interest.
