# Supplementary material for: Advanced Oxidative Protein Products Cause Pain Hypersensitivity in Rats by Inducing Dorsal Root Ganglion Neurons Apoptosis via NADPH Oxidase 4/c-Jun N-terminal Kinase Pathways
Source: Front Mol Neurosci. 2017 Jun 19;10:195. doi: 10.3389/fnmol.2017.00195 (PMC5474489; doi:10.3389/fnmol.2017.00195)
Supplement: Supplementary file 1 [file Data_Sheet_1.docx]

**Fig. S1**

**
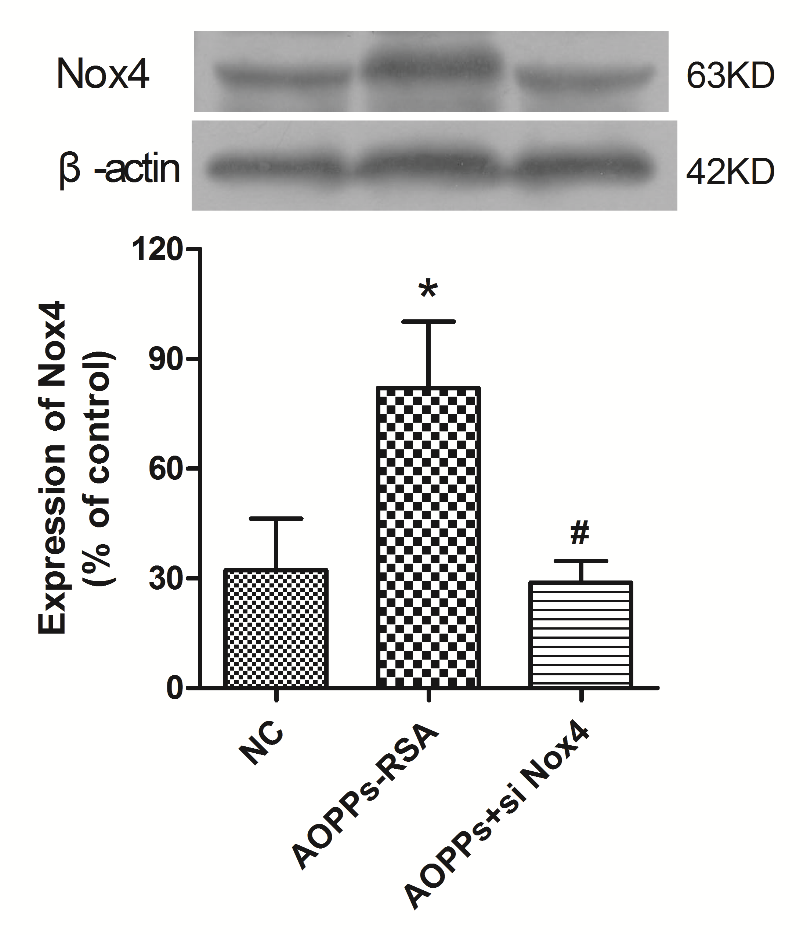
**

Fig. S1 After Nox4 siRNA pretreatment, cells were stimulated with or without AOPPs-RSA (200 μg/mL) for 6 h, bar graph showed quantitative analysis of Nox4. Data represent mean ± SEM of at least 3 independent experiments. * P<0.05 versus Control (0) group. # P<0.05 versus AOPPs-RSA group.

**Fig. S2**

**
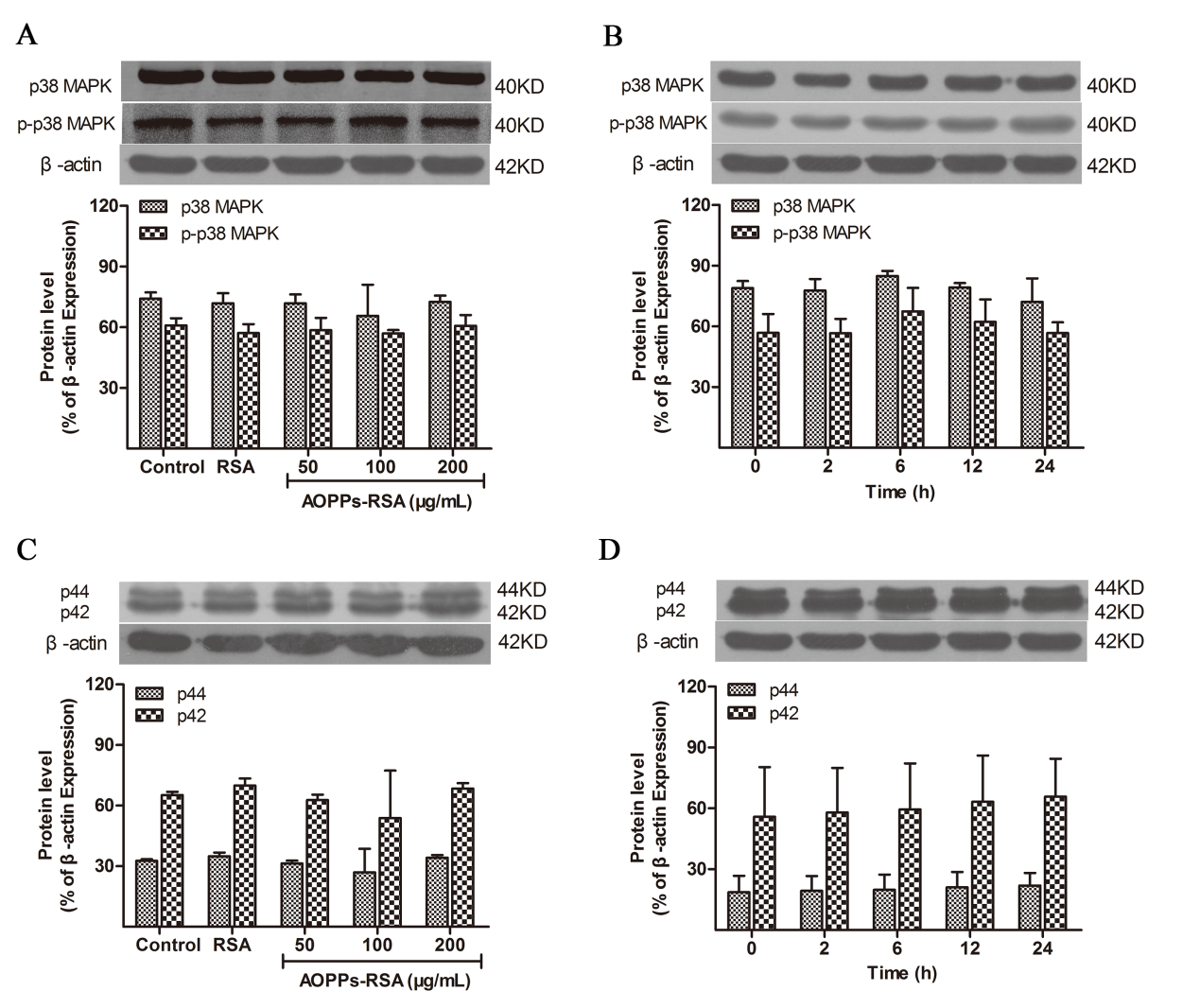
**

Fig. S2 The expression levels of p38/p-p38 and p44/p42. Data represent mean ± SEM of at least 3 independent experiments. * *P*<0.05 versus control (0) group. # *P*<0.05 versus AOPPs-RSA group.
